# The Demand for New Antibiotics: Antimicrobial Peptides, Nanoparticles, and Combinatorial Therapies as Future Strategies in Antibacterial Agent Design

**DOI:** 10.3389/fmicb.2020.01669

**Published:** 2020-07-24

**Authors:** Angel León-Buitimea, Cesar R. Garza-Cárdenas, Javier A. Garza-Cervantes, Jordy A. Lerma-Escalera, Jose R. Morones-Ramírez

**Affiliations:** ^1^Facultad de Ciencias Químicas, Universidad Autónoma de Nuevo León, UANL, San Nicolás de los Garza, Mexico; ^2^Centro de Investigación en Biotecnología y Nanotecnología, Facultad de Ciencias Químicas, Parque de Investigación e Innovación Tecnológica, Universidad Autónoma de Nuevo León, Apodaca, Mexico

**Keywords:** ESKAPE, MDR, XDR, antimicrobial peptides, metal nanoparticles, combinatorial treatments

## Abstract

The inappropriate use of antibiotics and an inadequate control of infections have led to the emergence of resistant strains which represent a major threat to public health and the global economy. Therefore, research and development of a new generation of antimicrobials to mitigate the spread of antibiotic resistance has become imperative. Current research and technology developments have promoted the improvement of antimicrobial agents that can selectively interact with a target site (e.g., a gene or a cellular process) or a specific pathogen. Antimicrobial peptides and metal nanoparticles exemplify a novel approach to treat infectious diseases. Nonetheless, combinatorial treatments have been recently considered as an excellent platform to design and develop the next generation of antibacterial agents. The combination of different drugs offers many advantages over their use as individual chemical moieties; these include a reduction in dosage of the individual drugs, fewer side effects compared to the monotherapy, reduced risk for the development of drug resistance, a better combined response compared to the effect of the individual drugs (synergistic effects), wide-spectrum antibacterial action, and the ability to attack simultaneously multiple target sites, in many occasions leading to an increased antibacterial effect. The selection of the appropriate combinatorial treatment is critical for the successful treatment of infections. Therefore, the design of combinatorial treatments provides a pathway to develop antimicrobial therapeutics with broad-spectrum antibacterial action, bactericidal instead of bacteriostatic mechanisms of action, and better efficacy against multidrug-resistant bacteria.

## Introduction

Development of antibacterial resistance is considered one of the leading public health problems, since it has a significant impact on the economy worldwide. Since therapeutic options to treat infections are increasingly being limited due to antibacterial resistance, this escalates the morbidity and mortality associated with infectious diseases caused by bacteria [World Health Organization (WHO), 2020]. ESKAPE pathogens are responsible for the majority of life-threatening nosocomial infections and are capable of “escaping” the biocidal action of antimicrobial agents (Pendleton et al., 2013). The term “ESKAPE” is an acronym for six bacterial pathogens associated with multidrug resistance: *Enterococcus faecium* (*E*. *faecium*), *Staphylococcus aureus* (*S*. *aureus*), *Klebsiella pneumoniae* (*K*. *pneumoniae*), *Acinetobacter baumannii* (*A*. *baumannii*), *Pseudomonas aeruginosa* (*P*. *aeruginosa*), and *Enterobacter spp.* (Mulani et al., 2019). Multidrug-resistant (MDR) bacteria are resistant to more than one antimicrobial drug, and extensively drug-resistant (XDR) bacteria are types of drug-resistant organisms that are resistant to all, or almost all, approved antimicrobial agents (Magiorakos et al., 2012). For these reasons, it is essential to design and engineer new promising classes of antibiotics (Gajdács, 2019).

## The New Therapeutic Alternatives: Input From Recent Studies

As we described above, the development of antimicrobial resistance represents a major threat to public health, and this has been echoed by different health organizations around the globe. Antimicrobial peptides (AMPs) and nanoparticles (NPs) and the design of novel combinatorial therapies are among the new promising alternatives to fight infections caused by MDR- and XDR-resistant bacteria.

### Antimicrobial Peptides

Antimicrobial peptides are a highly diverse family of small proteins with a varying number of amino acids; they have also been referred to as cationic host defense peptides (Boparai and Sharma, 2019). A variety of synthetic AMPs have been synthesized in the laboratories, but there are also a wide diversity of AMPs produced by bacteria and yeast, in addition to those found naturally in animals and plants (Wang, 2013). AMPs have demonstrated to participate in a variety of biological activities, including as antimicrobial antiviral, antifungal, and anti-mitogenic agents, in addition to their antitumor and anti-inflammatory properties and their ability to act as immune modulators. Therefore, AMPs represent a potential alternative to replace a wide variety of commonly used drugs. Moreover, most of the available studies demonstrate that AMPs exhibit therapeutic activity in *in vitro* and *in vivo* models (Divyashree et al., 2019).

**FIGURE 1:**
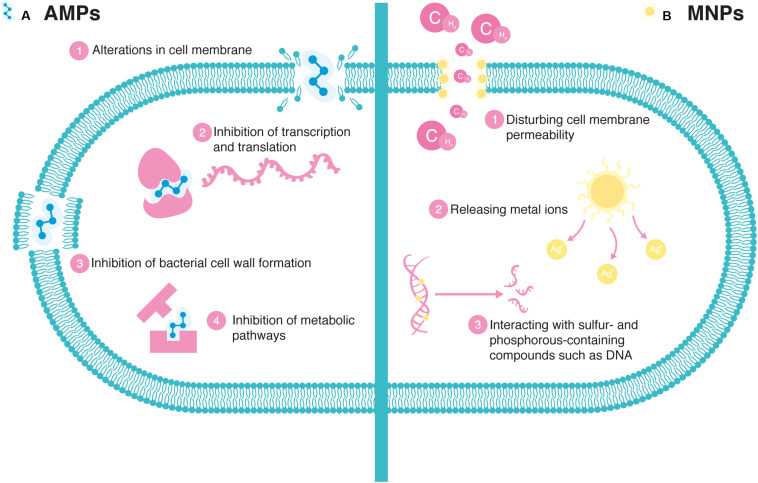
Mechanism of antimicrobial action in bacteria: **(A)** General mechanisms of action of antimicrobial peptides (AMPs). AMPs act through different mechanisms (Le et al., 2017) such as 1. Alteration in membrane integrity via electrostatic interaction with negatively charged cell membranes which kill cells, 2. Inhibition of DNA synthesis by (i) cross-linking with single- or double-stranded DNA, (ii) prevention of the DNA relaxation by inactivation of DNA topoisomerase I, and (iii) blocking of DNA replication by trapping the gyrase-DNA complex; and protein synthesis by (i) inhibition of protein translation by targeting the ribosomes, (ii) interrupting the protein-folding pathway, and (iii) rapid proteolytic activity causing the degradation of some DNA replication-associated proteins, leading to secondary inhibition of DNA synthesis, 3. Inhibition of bacterial cell wall formation by alteration of the alternating amino sugars in linear form that cross-link via peptide bridges to form the peptidoglycan layer, and 4. Inhibition of metabolic pathways by alteration of nucleic acid metabolism, including nucleotide transport and metabolism, nucleobase, nucleoside, and nucleotide interconversion. **(B)** Mechanisms for antimicrobial action of metal nanoparticles (MNPs). MNPs act via the following (Shaikh et al., 2019). 1. MNPs disturb cell membrane permeability by interfering with metabolic pathways and inducing changes in membrane shape and function. 2. When MNPs are in solution, metal ions are released in the environment surrounding. Metal ions generate reactive oxygen species (e.g., oxygen ions and hydroxyl radicals) and induce oxidative stress in bacteria. Oxidative stress is a key contributor in altering the bacterial membrane permeability and thus can damage cell membranes. Also, metal ions may cause cell structural changes and aberrant enzyme activities, which perturb normal physiological processes. 3. Interaction with sulfur- and phosphorous-containing compounds such as DNA, which prevent DNA from unwinding and transcription.

The use of AMPs alone or in combination with conventional drugs has proven effective in combating different infectious agents, mainly MDR bacteria (Zharkova et al., 2019). AMPs are promising potential candidates to counteract multiresistant pathogens since they possess many advantages: they display potent microbicidal activity in the micromolar range (Aoki and Ueda, 2013), they have demonstrated a rapid bacterial death action (Lei et al., 2019), and they have low resistance selection (Mahlapuu et al., 2016). Their mechanism of antibacterial action is multifunctional because it alters the cell membrane (Li et al., 2017) and also attacks specific targets that take part in the development of different intracellular processes (Le et al., 2017), such as inhibition of transcription, translation, protein synthesis, and bacterial cell wall formation (Mwangi et al., 2019). These general mechanisms of action of AMPs are displayed in Figure 1A.

One AMP of particular interest is human cathelicidin peptide (LL-37), which has been reported to have wound-healing effects on the host in addition to exhibiting antimicrobial and anti-biofilm activity against a variety of Gram-positive and Gram-negative human pathogens (Duplantier and van Hoek, 2013). LL-37 and its derivatives are considered excellent candidates as antimicrobial therapeutic agents and have been the subject of many studies (Dürr et al., 2006; Kościuczuk et al., 2012; De Breij et al., 2018). Especially, in 2018, De Breij et al. synthesized an LL-37 derivative (SAAP-148), with potent antimicrobial activities, by replacing an amino acid from the terminal carbon of the LL-37 chain. This LL-37 derivative exhibited a minimum inhibitory concentration [MIC] between 0.4 and 12.8 μM against various ESKAPE pathogenic bacteria (e.g., *E. faecium*, *S. aureus*, *K. pneumoniae*, *A. baumannii*, *P. aeruginosa*, and *Enterobacter* species) without selection of resistance. Furthermore, this AMP derivative showed anti-biofilm activity against *S. aureus*, *A. baumannii*, and *P. aeruginosa* (De Breij et al., 2018).

Colistin is another important peptide antibiotic (produced *Bacillus polymyxa* var. *colistinus*) used as a last-resort drug to treat MDR infections (Oka and Ito, 2000). It has emerged as an important agent in the treatment of Gram-negative bacterial infections, especially those caused by MDR pathogens in hospitalized patients (Das et al., 2017). Notably, two new colistin-derived AMPs (AA139 and SET-M33), with a mechanism similar to colistin, are in development and have shown excellent therapeutic potential both *in vitro* against MDR bacteria and in *in vivo* infection models (van der Weide et al., 2019).

The main limiting factor for the systemic use of AMPs is their sensitivity to proteolytic digestion in different body fluids (e.g., intestinal mucosa, gastrointestinal tract, and blood plasma), which directly affect both their *in vivo* stability and their pharmacokinetic profile (Moncla et al., 2011; Starr and Wimley, 2017). Therefore, the search for new AMPs continues, particularly in a new class of peptides with high specificity and potency, known as “selectively targeted AMPs” (STAMPs), which show increased sensitivity to specific pathogens, demonstrating a significant increase in their bactericidal capacity without direct effects on the microbiota (Chung and Khanum, 2017). The STAMP technology requires two functionally independent peptide domains integrated through a small linker. One peptide domain serves as the killing AMP moiety and the other peptide domain consisting of a high-affinity binding peptide which functions as a targeting moiety (Aoki and Ueda, 2013). These properties increase the binding to the surface of a targeted pathogen by enhancing the local concentration of the AMP and thus lead to improve bactericidal efficiency (Sarma et al., 2018). In recent years, several new and promising STEMs have been developed against *Streptococcus mutans* (Huo et al., 2017), *Pseudomonas aeruginosa*, and *Streptococcus mutants* together (He et al., 2009), methicillin-resistant *Staphylococcus aureus* (Mao et al., 2013), *Enterococcus faecalis* (Xu et al., 2020), and clinical isolates (*Pseudomonas aeruginosa*; Eckert et al., 2006). Nonetheless, more preclinical and clinical research is needed in the development of targeted antimicrobial therapy.

### Metal Nanoparticles

An additional alternative to fighting infections caused by antibiotic-resistant bacteria is the development of NPs since it has been amply reported that metal nanoparticles (MNPs) have antibacterial activity against ESKAPE pathogens (Wang et al., 2017; Lee et al., 2019). Some of the mechanisms of the antimicrobial mode of action of MNPs are summarized in Figure 1B. In the search for new antimicrobials to treat the ESKAPE pathogens, silver has been highlighted as a potential candidate to treat infectious diseases (Borthagaray et al., 2018). Silver nanoparticles (AgNPs) possess antimicrobial activity, and they act by disturbing cell membrane permeability, interacting with sulfur- and phosphorous-containing compounds including DNA, in addition to their ability to release silver ions, contributing to the antibacterial effect (Morones et al., 2005; Morones-Ramirez et al., 2013). Gold (Au) nanoparticles have also been reported as effective antibacterial agents for antibiotic-resistant bacterial strains such as *S*. *aureus*, *E*. *faecium*, *Enterococcus faecalis* (*E*. *faecium*), *Escherichia coli* (*E*. *coli*), *Vibrio cholerae* (*V*. *cholerae*), *Salmonella typhimurium* (*S*. *typhimurium*), and *Salmonella dysenteriae* (*S*. *dysenteriae*; Kumar et al., 2016).

Among metal oxide nanoparticles, zinc oxide (ZnO) nanoparticles have shown antimicrobial activity against both Gram-negative and Gram-positive bacteria, including *Bacillus subtilis* (*B*. *subtilis*), *S*. *aureus*, *E*. *coli*, *P*. *aeruginosa*, and *A*. *baumannii* (Guo et al., 2015; Tiwari et al., 2018). On the other hand, among photocatalytic nanoparticles, titanium dioxide (TiO_2_) NPs have been extensively studied due to their antimicrobial activity (Gelover et al., 2006). Several studies have reported the antimicrobial activity of TiO_2_ NPs against methicillin-resistant *S*. *aureus* and MDR *E*. *coli* (Jesline et al., 2015; Mantravadi, 2017; de Dicastillo et al., 2019).

Despite the advantages that nanoparticles offer, such as a broad therapeutic index, controlled drug release, less prone to bacterial resistance, and fewer side effects than chemical antimicrobials (Lee et al., 2019), to treat infections caused by the ESKAPE pathogens, there are still challenges remaining to be tackled such as improvement of physicochemical properties, better pharmacokinetic profiles, and comprehensive studies on long-term exposure to humans. In terms of design and application, there is a particular interest in the generation of nanohybrids combining different metals with different antimicrobial and sensitizer agents (Zhang et al., 2014; Wolfram et al., 2015). Metal nanoparticle-based compounds (alone or in combination with other antimicrobial agents) provide promising alternatives to combat the development of antibacterial resistance (Shaikh et al., 2019). Therefore, it is imperative to develop a comprehensive understanding of the mechanisms of action responsible for the bactericidal properties as well as the identification of the most promising antimicrobial agents for future clinical translation.

### Combinatorial Treatments

**FIGURE 2:**
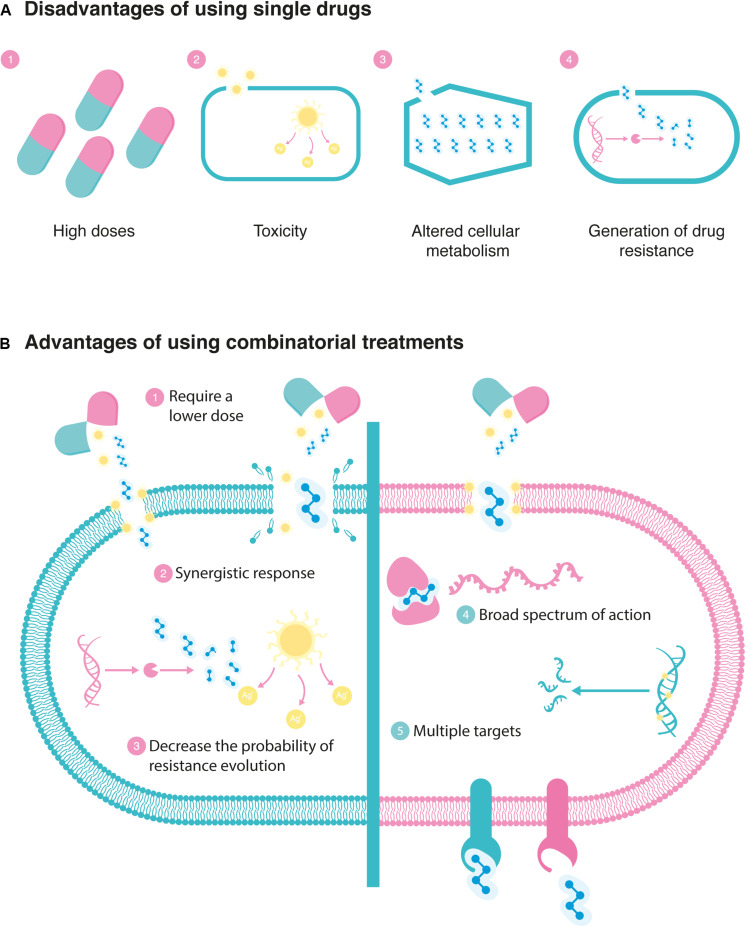
Antimicrobial treatment strategies: **(A)** disadvantages of using single drugs and **(B)** advantages of using combinatorial treatments. The advantage of using combinatorial treatments of synergistic drug pairs provides the opportunity to lower the dosage of the individual agents, thereby reducing toxicity while maintaining the wanted effect on bacteria. Moreover, a synergistic response can occur because of complementary drug action (multiple targets sites on the same protein or pathway are hit; Pemovska et al., 2018). By combining two drugs that achieve the same effect through different mechanisms of action, the development of resistance to a single drug in the combination may be less likely to occur, and when it does occur, it may have a lower impact on the therapeutic outcome (Pirrone et al., 2011). Finally, the use of more than one agent broadens the antibacterial spectrum of the empirical therapy and thus ensures that at least one agent will cover the infecting organism (Gurjar et al., 2014).

The strategies to reduce antibiotic resistance include the limited use of antibiotics and the application of more effective antibacterial therapies. Because the time of exposure to antibiotics correlates with the development of resistance (Andersson et al., 2020), it is necessary to use drugs with a broad spectrum of action and pharmacokinetic properties that facilitate their rapid access to the target site (Krause et al., 2016). However, most of the available treatments do not have all these characteristics, so an alternative option is the use of combination therapies, which can lead to a synergistic and more effective response (Lehár et al., 2009). It has been shown that the combination of drugs leads to a considerably more potent effect, compared to the individual drug (Tamma et al., 2012; Marks et al., 2013). Figure 2 displays the disadvantages of using single drugs (Figure 2A) and the advantages of using combinatorial treatments (Figure 2B).

#### Antimicrobial Peptide-Based Combinatorial Treatments

Combinations of AMPs with antibiotics have been reported to show synergistic effects in the treatment of bacterial infections. The mechanism of antibacterial action in these combinations involves the disruption of the outer membrane (Cassone and Otvos, 2010). Moreover, the use of AMPs in combinatorial treatments has certain advantages over their use as a single treatment since it has been observed that in combinatorial treatments, AMPs work as enhancers of the antimicrobial effects. This characteristic allows the reduction of their dose, and it also unlocks the bactericidal application of molecules with low molecular weight, which typically do not exhibit antimicrobial properties (Si et al., 2020).

Recent studies have demonstrated the synergistic activity of antibiotics combined with AMPs. Akbari et al. (2019) reported the synergism and other drug interactions between melittin, a cationic amphipathic peptide, and antibiotics such as doripenem, doxycycline, colistin, and ceftazidime, against MDR isolates of *A*. *baumannii* and *P*. *aeruginosa*. Likewise, combinatorial treatments of conventional antibiotics with new synthetic peptides inspired by human cationic peptides LL-37 and thrombocidin-1 (TC-1) have shown synergistic activity against *S*. *aureus* (antibacterial and anti-biofilm activity; Koppen et al., 2019). In addition, the synergistic activity of 30 short AMPs combined with several conventional antibiotics such as beta-lactam antibiotics, cephalosporins, aminoglycosides, and quinolones was tested against an MDR *P*. *aeruginosa* isolate (PA910; Ruden et al., 2019). Several combinations between peptides, polymyxin B, erythromycin, and tetracycline, as well as novel variants of indolicidin were found to be synergistic. Furthermore, the results showed that a single amino acid substitution within the peptides can have a powerful effect on the ability to synergize, which represents an opportunity to design treatment strategies based on synergistic interactions (Ruden et al., 2019).

#### Metal Nanoparticle-Based Combinatorial Treatments

Metal nanoparticles should be considered as an attractive alternative to potentiate the antimicrobial effect of old and current antibiotics, since they have a high tendency to act synergistically when combined with a wide variety of antibiotics (Bankier et al., 2019). This, in addition to the increased biocompatibility achieved by synthesizing them through green chemistry, allows considering the use of MNPs as adjuvant agents for the treatment of infectious diseases (Rout et al., 2018).

In the past years, there has been a marked increase in the use of biopolymers (e.g., proteins, nucleic acids, and polysaccharide) as capping agents to functionalize and stabilize MNPs (Sharma et al., 2019). Exopolysaccharides are biocompatible and eco-friendly biomolecules; therefore, they can be used in the synthesis of MNPs (Escárcega-González et al., 2018). Recently, a silver-based nanobiocomposite was synthesized using an exopolysaccharide produced by *Rhodotorula mucilaginosa* UANL-001L (EPS). The results showed an increased antibacterial and anti-biofilm activity of this nanobiocomposite against pathogens of clinical relevance (Vazquez-Rodriguez et al., 2020). Moreover, nanocomposites have been synthesized through green chemistry, such as zinc (Zn) and nickel (Ni) MNPs capped with EPS as capping agents, and they have displayed interesting antimicrobial properties as well. Ni-EPS nanoparticles exhibited both antimicrobial and anti-biofilm activity against resistant MDR strains of *S*. *aureus* and *P*. *aeruginosa*. Furthermore, Zn-EPS nanoparticles showed antimicrobial activity for treatments against MDR *S*. *aureus* and *P*. *aeruginosa* (Garza-Cervantes et al., 2019).

Among the most studied nanomaterials are silver nanoparticles due to their antimicrobial activity against Gram-positive and Gram-negative bacteria. They can be used in combinatorial treatments with currently used antibiotics for enhanced antimicrobial activity (Shahverdi et al., 2007; Kora and Rastogi, 2013; Naqvi et al., 2013; Singh et al., 2013; Panácek et al., 2016; Lopez-Carrizales et al., 2018; Perveen et al., 2018; Vazquez-Muñoz et al., 2019). Nonetheless, some other MNPs such as gold (El-Sheekh and El Kassas, 2014; Kalita et al., 2016; Al-Mawlawi and Obaid, 2019; Arya et al., 2019; Lee and Lee, 2019; Nishanthi et al., 2019; Yang et al., 2019), copper (Khurana et al., 2016; Woźniak-Budych et al., 2017; Murugan, 2018; Selvaraj et al., 2019), and zinc (Banoee et al., 2010; Bhande et al., 2013) have been used in combination with a variety of antibiotic families to enhance bactericidal efficacy.

Some other interesting studies of silver-based nanomaterials have been reported. A novel silver-microfibrillated cellulose biocomposite has been synthesized, and its antimicrobial activity was determined against relevant clinical strains. The results showed that this biocomposite has antimicrobial activity against Gram-negative and Gram-positive bacteria so that it could be applied in the development of biocompatible biomedical devices (Garza-Cervantes et al., 2020a). Another promising approach toward the development of new antimicrobial combinatorial treatments is the use of transition metals, since they exhibit rapid and significant toxicity, at low concentrations, in different bacterial strains. Garza-Cervantes et al. evaluated the synergistic antimicrobial effects of silver/transition-metal combinatorial treatments. Their results showed combinatorial treatments that exhibited synergism (Ag–Zn, Ag–Co, Ag–Cd, Ag–Ni, and Ag–Cu) since their antimicrobial effects are increased up to 8-fold, compared to the effects observed for the treatments with the individual metals (Garza-Cervantes et al., 2017). Furthermore, Montelongo-Peralta et al. reported synergism between transition metals and antibiotics used to treat first-line drug-resistant strains of *Mycobacterium tuberculosis* (*M*. *tuberculosis*). Combinatorial treatments composed of isoniazid/silver exhibited a synergistic bactericidal effect in an isoniazid-resistant clinical strain of *M*. *tuberculosis* (Montelongo-Peralta et al., 2019).

Moreover, a previous study showed the ability of silver to potentiate the activity of a broad range of antibiotics against Gram-negative bacteria, as well as to restore antibiotic susceptibility (re-sensitizing) to a resistant bacterial strain (Morones-Ramirez et al., 2013). Recently, a group of researchers achieved to re-sensitize antibiotic-resistant *E*. *coli* using transition-metal micronutrients (Cu2+, Zn2+, Co2+, Cd2+, and Ni2+) combined with antibiotics (ampicillin and kanamycin). These combinatorial treatments showed a therapeutic activity and no toxicological effects in a murine topical infection model caused by antibiotic-resistant strains (Garza-Cervantes et al., 2020b). The above data therefore strongly suggest that combination therapies are a potential strategy in the development of new treatments against infectious diseases.

The search for a new generation of antimicrobials to mitigate the spread of antibiotic resistance is urgent (de la Fuente-Nunez et al., 2017). Current research and technology developments have promoted the improvement of antimicrobial agents that selectively target a target site (e.g., a gene, a cellular process, or a specific pathogen; de la Fuente-Nunez et al., 2017; Jackson et al., 2018). AMPs and MNPs exemplify a novel approach for treating infectious diseases. Nonetheless, the combinatorial treatments are considered as an excellent option for designing and developing next-generation antibacterial agents. As summary, Table 1 describes the mechanism of action, tested bacterial strains, advantages and disadvantages of AMPs, MNPs, and combinatorial treatments.

**TABLE 1:** Antimicrobial peptides, metal nanoparticles, and combinatorial treatments: mechanism of action, tested bacterial strains, advantages, and disadvantages.

	**Mechanism of action**	**Tested bacterial strains**	**Advantages**	**Disadvantages**
Antimicrobial peptides (AMPs)	1. Alteration in membrane integrity. 2. Inhibition of DNA and protein synthesis. 3. Inhibition of bacterial cell wall formation. 4. Inhibition of metabolic pathways.	*Enterococcus faecium*, *Staphylococcus aureus*, *Klebsiella pneumoniae*, *Acinetobacter baumanii*, *Pseudomonas aeruginosa*, *Enterobacter spp.*, multidrug-resistant strains.	1. Show potent microbicidal activity in the micromolar range. 2. Rapid bacterial death action. 3. Low resistance selection.	1. High sensitivity to proteolytic digestion in different body fluids. 2. Low *in vivo* stability. 3. Reduced pharmacokinetic profile.
Metal nanoparticles (MNPs)	1. Disruption of cell membrane and increased permeability. 2. Releasing metal ions. 3. Interaction with DNA	*Enterococcus faecium*, *Enterococcus faecalis, Staphylococcus aureus*, *Klebsiella pneumoniae*, *Acinetobacter baumanii*, *Pseudomonas aeruginosa*, *Escherichia coli*, *Salmonella typhimurium*, *Salmonella dysenteriae*, *Vibrio cholerae*, *Bacillus subtilis*, multidrug-resistant strains.	1. Broad therapeutic index. 2. Controlled drug release. 3. Less prone to bacterial resistance. 4. Fewer side effects than chemical antimicrobials.	1. Need to improve metal ions release from MNPs. 2. Moderate stability in biological fluids. 3. Reduced long-term toxicity studies.
Combinatorial treatments	1. Synergistic response. 2. Multiple cellular targets for antimicrobial action. 3. Combination of bactericidal and bacteriostatic mechanism of action.	*Enterococcus faecium*, *Staphylococcus aureus*, *Klebsiella pneumoniae*, *Acinetobacter baumanii*, *Pseudomonas aeruginosa*, *Escherichia coli Mycobacterium tuberculosis* multidrug-resistant strains.	1. Require lower dose than a single drug. 2. Reduced toxicity. 3. Synergisms and more effective response. 4. Decrease the probability of resistance evolution. 5. Better efficacy against multidrug-resistant bacteria.	1. Physical-chemical compatibility among antimicrobial agents. 2. Possible pharmacokinetic and pharmacodynamic interactions.

The selection of appropriate combinatorial treatment is critical for the successful prevention of infections (Bayramov and Neff, 2017). The most important challenges include (i) selection of agents with ideal physical–chemical properties (hydrosolubility and chemical stability in biological fluids; Ebejer et al., 2016), (ii) selection of antimicrobials that display appropriate pharmacokinetics and pharmacodynamics properties (Preston, 2004), (iii) selection of biocompatible capping agents or biopolymer-based materials that enable drug release (Campoccia et al., 2013), and (iv) development of a process that ensures the stability and does not compromise the performance of the combination therapy formulation as a whole (Wu and Grainger, 2006). Therefore, the design of combinatorial treatment provides a pathway to develop antimicrobial therapeutics with broad-spectrum antimicrobial activity, bactericidal instead of bacteriostatic mechanism of action, and better efficacy against MDR bacteria.

## Author Contributions

Conceptualization, AL-B and JM-R. Writing-original draft preparation AL-B, CG-C, JG-C, JL-E, and JM-R. Graphic design, JL-E. Writing-review and editing, AL-B, CG-C, JG-C, and JM-R. Supervision, AL-B and JM-R. All authors contributed to the article and approved the submitted version.

## Conflict of Interest

The authors declare that the research was conducted in the absence of any commercial or financial relationships that could be construed as a potential conflict of interest.
